# The survey visit as a key evaluative event in accreditation–a qualitative study of survey visit experiences among surveyors and general practice professionals

**DOI:** 10.1186/s12875-021-01497-7

**Published:** 2021-07-31

**Authors:** Tina Drud Due, Thorkil Thorsen, Marius Brostrøm Kousgaard

**Affiliations:** The Research Unit for General Practice and Section of General Practice, Department of Public Health, University of Copenhagen, Øster Farimagsgade 5, 1014 Copenhagen, Denmark

**Keywords:** Accreditation, Survey visit, Compliance, Assessment, Standards, Primary care, General practice

## Abstract

**Background:**

Accreditation is a widely employed quality assurance concept in health care and the survey visit is the central method for assessing participating organisations’ compliance with accreditation standards. Despite this, research on the survey visit as a method for assessing compliance is scarce. In Denmark a mandatory accreditation programme was introduced for general practice clinics in 2016. We performed a qualitative, explorative study of the reflections and actions of surveyors and general practice professionals (GPs and staff) concerning the production of information about compliance with the accreditation standards in relation to the survey visit.

**Methods:**

We conducted qualitative interviews with GPs and staff from general practices in two Danish regions before and after their survey visit. We also interviewed the surveyors. We observed survey visits to qualify the interviews and analysis. All interviews were audio recorded, transcribed, and analysed using an integrative approach.

**Results:**

The surveyors combined documents, questioning of the professionals, and visual impressions of the clinic to assess compliance. They sought to de-dramatise the survey visit and to generate a natural conversation with attention to workflows. Trust in the professionals’ statements was fundamental to the surveyors’ approach, and they were confident in their ability to assess compliance. Their level of scrutiny was influenced by their observations and the quality of documents. The general practice professionals had generally sought to comply with the standards and to give an authentic portrait of the clinic. The few cases of misrepresention concerned standards that the professionals found too excessive.

**Conclusion:**

The validity of the survey visit as a method to assess compliance was highly dependent on the professionals’ willingness to convey a realistic picture of their practice. Since they were generally willing to do so, the trust-based approach seemed suitable for identifying cases of non-compliance caused by insufficient understanding of the standards. However, it can be difficult for the surveyors to detect when the professionals engage in misrepresentation due to disagreements with the standards. Thus, when adopting a trust-based approach to the survey visit, it seems particularly important to ensure that the professionals view the standards as meaningful and manageable.

**Supplementary Information:**

The online version contains supplementary material available at 10.1186/s12875-021-01497-7.

## Background

Accreditation is a widely employed concept for quality assurance in health care. The basic principle of accreditation is the external assessment of health organisations against a set of predefined standards for quality and patient safety [1]. While traditional models of health care accreditation were characterised by being ‘voluntary, confidential and self-financed’ [2], accreditation has gradually become a more regulatory tool characterised by increased government involvement, transparency, and demands of mandatory participation [1–3].

In accreditation, the survey visit – a visit performed by surveyors from the accreditation institution to the participating organisations – is the central method for assessing whether the participating organisations comply with the accreditation standards. Despite this, there is little research on the survey visit as a method for assessing compliance. Some studies have focused on inter-surveyor reliability (i.e. on consistency in assessments between surveyors) and the elements affecting it, such as the construction and understanding of the standards, the training of surveyors, and the timeframe of the survey visit [4–7]. However, a related and equally important aspect concerns the validity of the survey visit as an assessment method, i.e. whether the survey visit allows the surveyors to obtain a realistic impression of the organisations’ level of compliance with the standards. This depends on the factual accuracy of the information on which the surveyors ground their assessment in individual cases. This information is usually comprised by documents prepared by the participating organisations, the statements of the professionals during the survey visit, and on observations made by the surveyors during the visit [6]. This implies that the survey visit as an assessment method relies on a certain level of trust in the actors being assessed as well as on the surveyors’ efforts and opportunities in regards to verifying the descriptions presented by the members of the assessed organisations. However, from a regulation perspective, organisations’ compliance with external rules and standards cannot be taken for granted since non-compliance may be caused by several factors such as lack of knowledge or understanding of the requirements, lack of ability or capacity to comply, and lack of willingness to comply due to cost considerations or disagreement with the requirements [8]. And if the regulated organisations are not interested in complying with the standards, a high level of dependence on the information supplied by the organisations could constitute a challenge to the assessment. Thus, research on organisational responses to external performance measurement suggests that organisations may sometimes deliberately manipulate reported information so that reported behaviour is inconsistent with actual behaviour, a phenomenon termed *misrepresentation* [9, 10]. In the case of accreditation, this could mean that organisations that are not interested in complying with the standards, but still wish to gain accreditation approval, could attempt to misrepresent their organisational practice to the surveyors, so that areas of non-compliance are not identified. A study among accreditation stakeholders in Australia found that if participating organisations had a difficult relationship with the accreditation agency and the survey team, this could lead them to withhold information from the survey team [6]. However, the study did not elaborate further on this issue, and generally little is known about the experiences and actions of surveyors and health professionals in relations to such aspects of the accreditation survey.

On this background, we performed a qualitative, explorative study of the reflections and actions of surveyors and general practice professionals (GPs and staff) concerning the production of information about compliance with the accreditation standards in relation to the survey visit. The survey visits were carried out as part of a mandatory accreditation programme for general practice, which was implemented in Denmark from 2016–2018.

## Setting and intervention: accreditation in Danish general practice

Danish health care is mainly tax financed with free-of-charge access to general practice and public hospitals. General Practitioners (GPs) are private entrepreneurs mostly financed through the public health care reimbursement scheme and services are regulated by collective agreements between the Danish Regions and the Organisation of General Practitioners [11]. Danish general practice is divided into 42% solo-clinics and 58% partnership clinics co-owned by two or more GPs (the latter covering 79% of the GPs) [12].

Accreditation was mandatory (except for clinics intended to close within five years) and implemented for the first time in Danish general practice in 2016–18. After all participating clinics had been through the accreditation process, the accreditation programme was terminated (and replaced with a new model for quality improvement based on so-called quality clusters). The Danish Institute for Quality and Accreditation in Healthcare (IKAS) was the institution responsible for carrying out accreditation. The standard set was constructed by IKAS and representatives from the Danish Regions, the Organisation of General Practitioners in Denmark, the Danish College of General Practitioners, the Danish Association of Practicing Medical Specialists, and Danish Patients. The accreditation standards covered 16 standards with 64 associated indicators. The topics of the standards are shown in Table 1. The standards generally stated overall requirements and provided references to guidelines and other documents to identify the more detailed requirements. Most indicators required that the professionals could account for their work processes at the survey visit. Some standards required these descriptions to be textual while for other standards, oral accounts of workflows were sufficient.

**Table 1:** The 16 standards of the accreditation programme

Standard	Standard content
1. The professional quality	Use of diagnosis coding Collection, analysis and use of clinical data for quality improvement
2. Use of good clinical practice	Detection, course of treatment and division of labour between GP and staff for patients with diabetes or COPD and for vulnerable patients
3. Adverse events	Reporting, follow-up and process for learning in case of adverse events
4. Patient evaluations	Completion of a patient evaluation via DAK-E and follow-up on the results
5. Prevention of confusion of patient’s identity	Identification of patients principally by social security number and labelling of diagnostic material
6. Prescription of medicine and renewal of prescriptions	Rational and safe medicine ordination and renewal of prescriptions Participation in regional initiatives for correct medicine management Annual assessment of patients’ list of medicine Reporting of side effects
7. Paraclinical tests	Execution of tests and handling of test materials Quality control of equipment Requisition and follow-up of paraclinical tests Procedures for test results in case of GP’s absence Procedures for missing tests results
8. Emergency response and cardiac arrest	Handling of acute disease and cardiac arrest in the clinic Participation in cardiopulmonary resuscitation course
9. The patient health record, data safety and confidentiality	Content of patient health record conforms to current legislation Journal audit performed and follow-up upon if needed Safe storage, handling and destruction of sensitive personal data Discretion and confidentiality in patient contacts
10. Availability	Accessibility in accordance with collective agreement (e.g. telephone hours, opening hours and waiting time) Physical accessibility Visitation of patients Online practice declaration with relevant information
11. Referral	Relevant and adequate content and handling of referrals
12. Coordination of patient care	Coordination and continuity of internal patient trajectories and externally with other health care providers
13. Acquisition, storage and disposal of clinical utensils and medicine/vaccines	Sufficient stuck of utensils, medicine and vaccines Correct storage of medicine e.g. at the right temperature Control of expiration dates Correct disposal
14. Hygiene	Cleaning of the clinic and inventory Cleaning and storage of medical equipment Correct hand hygiene Management of infectious patients
15. Management and operational activities	Ensure clear leadership, resource optimisation and development by having a plan containing plans for quality improvement, division of responsibilities and tasks, quality monitoring of e.g. patient records, equipment and medicine, and development of the clinic and development goals
16. Hiring, introduction and competency development	Ensure correct competences when hiring Introduction of new GPs, GPs in training and staff Supervision and competency development

The clinics were notified one year before their scheduled survey visit. The survey visit was conducted by two surveyors who questioned the GPs and the staff to determine whether the clinic complied with the accreditation standards. One surveyor was a GP (active or retired), and the staff-surveyor had a background in health care and often experience from general practice (e.g. nurse). The survey visit was scheduled to last for about 4 h in solo clinics and extra hours could be added in clinics with more GPs.

The surveyors had three different data sources available in their assessment: a) documents composed by the clinics prior to the survey visit that accounted for their work processes and how they complied with the standards, b) oral statements of the professionals during the survey, and c) visual impressions of the clinic during the survey. In IKAS’ value statement it was noted that the surveyors should be stringent but avoid pedantry and give room for different solutions to following the purpose of the standards. IKAS also emphasised the importance of the surveyors meeting the clinics with a fundamental sense of trust:

*“Basically, the surveyors shall meet the institutions to be accredited with trust: We believe in what we are told, unless there is reason not to, and in return we expect honest answers” (IKAS’ webpage *[13]*).*

After the survey, the clinics received the surveyors’ summarised report to which they could make objections in case of misunderstandings. Subsequently, the accreditation agency decided on the granting of accreditation status: accredited, accredited with remarks, not accredited. Clinics that did not meet the accreditation standards had to go through a follow up process (via phone or an additional survey) where they had to demonstrate compliance in order to receive accreditation. Accreditation results were published on IKAS’ website. In case clinics were not accredited IKAS would report it to the Danish Patient Safety Authority, Danish Regions, and the Organisation of General Practitioners. When the accreditation programme was initiated the question of the consequences of not being accredited was not settled. At the end of the accreditation programme 1606 clinics had been through the accreditation process; 1596 (99%) were accredited (hereof 34 with remarks), and only 10 clinics were not accredited. Each clinic received 20.000 Danish kroner (approx. £2300) per GP in the clinic for their participation (half of the amount was paid in advance and the rest when the clinic had achieved accreditation).

## Methods

### Qualitative interviews

The study consisted of qualitative interviews with professionals from general practice and with surveyors. The professionals from general practice included GPs and their staff (nurses and secretaries) and they were recruited from general practices (set to receive survey visits in 2017) in two Danish regions: The Capital Region and Region Zealand. The clinics were strategically sampled [14] based on geography, clinic type (solo/partnership) and a priori attitudes towards accreditation stated in a previous survey [15]. Since, general practice in Denmark had not been accredited before, we deemed it important to interview the clinics twice: The first interview was conducted 3–8 months before the survey visit as the clinic was preparing for the visit; the second interview was conducted 2–7 month after the survey visit. For this study, the first interview gave insight into the professionals’ expectations about the survey visit and their motivations and actions prior to the survey, and the second interview explored their experiences with the actual survey visit and how they acted during and after the visit. GPs and staff were interviewed separately, and each interview lasted about one hour. Originally, 12 clinics were included in the study, but one clinic was later excluded due to postponement of its survey date. Information on the interviewees and their clinics is presented in Table 2.

**Table 2:** Clinics and interviewees in the study

Clinic	Clinic type	GPs and staff	1. round interview participants	2. round interview participants	A priori attitude to accreditation^b^	Survey visit observed
1	Partnership	3 GPs, 1 nurse, 2 secretaries	2 GPs, 1 nurse, 1 secretary	1 GP, 1 nurse, 1 secretary	Negative	Yes
2	Solo	1 GP, 2 nurses	1 GP, 2 nurses	1 GP, 2 nurses	Positive	Yes
3	Partnership	3 GPs, 2 nurses, 3 secretaries	3 GPs, 2 nurses, 1 secretary	3 GPs, 2 nurses, 1 secretary	Negative	Yes
4	Solo	1 GP, 1 biomedical laboratory scientist	1 GP, 1 biomedical laboratory scientist	1 GP, 1 biomedical laboratory scientist	Positive	Yes
5	Solo	1 GP, 1 secretary	1 GP, 1 secretary	1 GP, 1 secretary	N.A	No
6	Partnership	3 GPs, 3 nurses, 1 secretary	3 GPs, 2 nurses, 1 secretary	3 GPs, 2 nurses, 1 secretary	Positive	Yes
7	Solo	1 GP, 1 nurse	1 GP	1 GP	Negative	Yes
8	Partnership	2 GPs, 2 nurses	2 GPs, 2 nurses	2 GPs, 2 nurses	Negative	Yes
9^a^	Partnership	2 GPs, 1 secretary	2 GPs		Positive	Yes
10	Solo	1 GP, 1 nurse	1 GP, 1 nurse	1 GP, 1 nurse	Negative	Yes
11	Solo	1 GP, 1 nurse	1 GP, 1 nurse	1 GP, 1 nurse	Positive	Yes
12	Partnership	3 GPs, 2 nurses, 2 secretaries	3 GPs, 2 nurses	3 GPs, 2 nurses	Negative^c^ Positive	Yes

^a^Survey visit postponed; clinic excluded from the study

^b^Based on a survey conducted before the initiation of the programme

^c^Two different GPs had answered the questionnaire

Furthermore, we interviewed the surveyors who had conducted the surveys in the included clinics. We interviewed 4 GP-surveyors and 6 staff-surveyors (Table 3). One of the GP-surveyors was not able to participate in an interview. The interviews with the surveyors also lasted approximately one hour.

**Table 3:** Interviewed surveyors

Surveyor	Gender	Clinic visited
GP-surveyor 1	M	1 and 12
GP-surveyor 2	F	3 and 4 and 10
GP-surveyor 3	M	5
GP-surveyor 4	M	7 and 8 and 11
GP-surveyor 5 (not interviewed)	M	2 and 6
Staff-surveyor 1	F	1
Staff-surveyor 2	F	2 and 5 and 10
Staff-surveyor 3	F	6 and 8
Staff-surveyor 4	F	4 and 7 and 12
Staff-surveyor 5	F	3
Staff-surveyor 6	F	11

As a step in developing the interview guide, we first carried out pilot interviews in two clinics that had already been accredited. We also observed the survey visits in all but one of the interviewed clinics to qualify the interview guides (generally and in terms of posing specific questions to each clinic) and to increase our knowledge of the survey visit. The topics in the interview guides relevant for this paper are listed in Table 4 and a full translation of the interview guides are presented in Supplementary file 1.

**Table 4:** Topics in the interview guides

Interview guide for the clinics
Description of the survey and how it matched their expectations
Experiences with and reflections on the surveyors’ approach to assess compliance with the standards and ability to obtain an adequate and valid assessment
The form of communication at the visit
Descriptions and reflections on what they told and showed the surveyors
Opinions about the surveyor being a colleague
Thoughts about potential remarks and the process after the survey (continued adherence or de-implementation)
Interview guide for the surveyors
Preparation before the visit
Their approach and techniques during the survey
Their reflections on the professionals’ opportunities for not representing an authentic image of their clinic
Their role as a representative from the accreditation agency while also being a colleague
The work division between the GP-surveyor and the staff-surveyor
Specific questions prompted by our observations of survey visits

We emphasised to all the interviewees that we as researchers had no affiliation to IKAS, the Regions, or other stakeholders, and that we had no interests in specific study results. Furthermore, all interviewees were promised anonymity and we emphasised that no identifiable information would be provided to IKAS or other stakeholders.

### Analysis

All interviews were audio recorded and transcribed. We adopted an integrative approach to the analysis by combining an inductive with a more deductive approach to developing codes and themes [16]. First, we read the interviews to increase familiarity with the data and identify potential domains and themes [16, 17]. In this process we also integrated some of the pre-defined topics from the interview guide, and hereby we developed an initial coding structure. With this coding structure we all coded one surveyor interview and the four interviews from one of the clinic (GPs’ and staff’ pre and post survey interviews) and compared and discussed our coding and adjusted the coding structure accordingly. Subsequently, we coded the rest of the interviews. Based on an analysis of the coded extracts we constructed and summarised the primary domains (e.g. the surveyors’ approaches to the survey visit, the surveyors’ use of specific techniques, the clinics’ approaches to the survey visits, issues of misrepresentation, the surveyor being a colleague). We re-read individual interviews in case of doubts or if elaborations were needed. We then identified and discussed themes within or across the different domains (e.g. trust as a permeating feature of the surveyors’ approach; surveyors’ confidence in own performance, the collegial status of the surveyors as an asset; the professionals generally seeking to comply with the standards; and misrepresentation as a rarely used tactic mainly driven by disagreement with the standards). The analysis proceeded by focusing on themes that needed further unfolding and explaining.

As social scientists, we had an outsider’s view and no vested interests in the study result. During interviews and analysis, we were conscious of and challenged our pre-conceptions, which included an uncertainty about the surveyors’ ability to obtain a valid assessment during the survey visit and our awareness that many GPs were negative about accreditation.

## Results

### The surveyors’ approach, experiences, and reflections

#### De-dramatising the survey visit

Since the surveyors were aware that some professionals in the clinics could be nervous, frustrated and/or critical towards the survey visit, they were attentive to establishing a pleasant atmosphere and to dedramatise the situation both prior to and during the visit. They experienced that a telephone call prior to the visit, and a joint session at the beginning of the visit where the surveyors and the professionals introduced themselves to each other combined with a tour of the clinic, contributed to this. A staff-surveyor also described how the dialogue and time for a bit of chitchat and a laugh also loosened up the professionals’ pleasure in narrating. Further, the surveyors believed that being colleagues was a mitigating factor, which took the sting out of some profesionals’ frustrations about accreditation, and that being collegues was part of the reason why they were usually met with an open and respectfull attitude, also by professionals who beforehand had negative attitudes towards the accreditation programme.

#### Quality of documents influencing the level of scrutiny

The surveyors experienced that the clinics were generally well prepared for the visits. Most clinics they visited and all clinics in this study had uploaded their documents to IKAS prior to the survey visit (although it was only required that they could present them at the visit). Some had even prepared documents on standards that only had to be accounted for orally. The surveyors experienced that this facilitated their own preparation and a better use of the time during the visit. The surveyors used the uploaded documents to form an overall impression of the clinic and to find out if there were areas that were not well described or appeared not to comply with the standards. Although some surveyors mentioned that they also asked into areas that were well described in the documents in order to assess consistency between the documents and the answers given at the visit, most surveyors’ way of inquiring was influenced by the content of the documents. Hence, during the survey visit they paid more attention to areas that appeared to be ‘weak’ in the documents, whereas well described areas were less meticulously covered with either no or few affirmative questions and observations.

*“I check whether they meet the requirements present in the standards […] and I skim-read the rest to get a feeling beforehand of what kind of clinic it is, so that when I leave for the clinic, I usually know almost how it will turn out and how it is, you know. It gives me a sense of where I should have a little extra focus, and if they say ‘we do it like this and like this and so on’, and this is also what they have written, I promptly know that that’s how it is” (GP surveyor #2).*

#### Relating the interview to the various space(s) of the clinic

After the presentation at the joint session, the surveyors engaged in a talk about those standards that were common for the GPs and staff. Then they split up so that the GP-surveyor interviewed the GPs in their consultation room and the staff-surveyor interviewed the staff. The surveyors explained how they preferred to interview the professionals about the standards in the room where the relevant process was taking place during everyday work. For the staff-surveyors this meant that they often walked around in the clinic while they interviewed the staff, so that the questions and answers could be related to specific locations and specific instruments and materials:

*“I always walk around; I don’t sit down that much with the staff because the staff is not used to sit still. They are used to moving around in the clinic […]. And it also makes them more calm that the things we talk about make sense in the room we are in. So, when we talk about laboratory matters, we are in the lab. And then they show me the different procedures they perform. ‘How do you check your autoclave?’ ‘How do you use the dishwasher?’ ‘How do you do these things?’ ‘Have you ensured… how do you check your utensils and your medicine, vaccines etc.?’ ‘Do you keep a log, and how do you do it?’” (Staff-surveyor #5).*

#### Striving for an open and natural conversation

The surveyors described how they used a combination of concrete questions, follow-up questions, process-questions (asking the professionals to describe a short workflow e.g. process of cleaning medical utensils including products used, and monitoring of equipment quality), and so called ‘tracer-questions’ intended to cover several standards in a more narrative manner (making the professionals describe the whole process from when a certain type of patient enters the clinic and through all the different interactions, tests and follow-ups). The surveyors strove to facilitate an open and natural conversation. They did not ask slavishly into the standards one by one, because in their experience this appeared artificial and too much like an examination. Instead, they would let the dialogue lead the flow of topics and standards covered. They also experienced that using variants of the tracer questions and process questions enhanced the professionals’ tendency to talk more openly and give more credible answers. Further, they emphasised that they were colleagues and occasionally drew on their own experiences from general practice (including their own accreditation process). A staff-surveyor explained how she told the professionals about events from her own clinic in order to make the situation less formalistic and loosen up the conversation if they appeared nervous.

#### Visual observations influencing the level of scrutiny

The surveyors used visual observations in two ways during the visit: a) to form a general impression of the clinic and b) to help them in assessing the clinics’ compliance with specific standards. Like the content of the documents influenced the surveyors’ focus in regards to questioning and visual observations, the visual observations also guided the direction and intensity of their questioning, so that areas which appeared to be less adequate would receive increased attention and questioning and vice versa:

*“If I visit at clinic where I get the impression [e.g. from the number of available spirit dispensers] that the staff washes their hands with spirit when they do different things, then I don’t ask about when they wash their hands with spirit or soap and when they do both; I would find it a bit inappropriate. But when visiting a clinic and finding at the doctor’s desk a hand disinfectant from the year 2011, and it is completely cloudy, the uh… then it perhaps seems reasonable to inquire into it” (Staff-surveyor #2).*

The staff-surveyors used their opportunities for visual assesments frequently; they asked into the cleaning procedures in the room where this took place and where they could see the equipment used; some of them looked at the clinics’ log-schemes and calenders for control of equipment, refrigerator temperature and medicine; some checked documentation for attending courses in cardiopulmonary resuscitation, and some also checked the storage and expiration dates of medicine and utensils. Again, there were variations, with some surveyors mainly asking into the procedures and others also using the visual opportunities:

*“Well, we always have a tour around the clinic… and I as a staff-surveyor I also get around in the clinic. I see their different procedures. I see where they keep their utensils and medicine and the like. And thus, you quickly get an impression as to whether the clinic is clean and orderly and whether they keep things correctly, and discretion and so on” (Staff-surveyor #3).*

A staff-surveyor experienced that making use of the opportunities for observation at the survey visit was important since this could help uncover situations where the descriptions in the documents were not accurate. Here, she recalls an example from one of her visits:

*“Especially when I was with the nurse and the secretary and we started to open some cupboards and saw that there wasn’t that much systematics in how things were organised. The acute medicine was placed a bit at random. Disorganised. Therefore, I realised that it wasn’t as clear cut as it was originally stated in the clinic’s documents” (Staff -surveyor #6).*

As part of the assessment, the GP-surveyors asked the GPs to show them a number of patient records for patients with diabetes and COPD. Some GPs had selected them in advance of the visit and the surveyors found this to be acceptable although some believed that the GPs had selected favorable cases. Others trusted that the selected records illustrated the general approach in the clinic. The surveyors recounted that they had also found flaws in patient records selected in advance.

The surveyors usually looked at three to four patient records during a visit. They did not consider this a represenative sample but rather as a departure point for the interview, or as a way to form an overall picture of the level of systematic chronic care in the clinic:

*“You see, it is the overall picture […]. Because if they say that here is an isolated case where it hasn’t been done, and then if there is also something else, one thing here and one thing there, then you start to become suspicious. Because suddenly there are many cases. Then we can’t describe it all as isolated cases […] Then you sharpen your attention” (GP-surveyor #1).*

When assessing the records, the surveyors considered patient attendance to annual disease check-ups, updating of the shared medication record, referrals and discharge letters, and if they as a locum would be able to understand the records and treat the patients based on the records. The level of details in this assessment seemed to vary between the surveyors. Further, some explained that they initially used a few self-selected indicators (like an updated shared medication record or use of standard phrases in the records) to gain an impression of the level of structured care in the clinic, and that this impression influenced the level of detail in their further inquiry. Hence, they looked at fewer records in case these areas were in order in the first records, and contrary it resulted in more stringent attention if they were not:

*“…three or four records. More if there are problems. Then they get the chance 'couldn't you just show me one more'. And if I can just see that both this and that is in order, and they use standard phrases and so on then I just look at a few and then it's fine” (GP surveyor #2).*

Again, this quote illustrates that the records were used more to form an impression and inspire a dialouge than to perform a systematic control for compliance.

#### Trust in the professionals and confidence in own ability to assses compliance

The surveyors believed they covered all standards and indicators sufficiently and that they obtained a valid picture of the clinics’ level of compliance. In five of the clinics in the study, the surveyors identified and gave remarks on noncompliance with the standards (Table 5).

**Table 5:** Cases of non-compliance with the standards identified by the surveyors in the 11 clinics

Standard	Remark
1. The professional quality	Insufficient use of diagnosis coding (Clinic 5)
2. Use of good clinical practice	Had not selected a specific vulnerable group and made a related procedure for managing them (Clinic 2 and 5)
4. Patient evaluations	Had not yet performed (or finished) the audit of patient records (Clinic 5 and 11)
5. Prevention of confusion of patient’s identity	Did not identify all patients by asking for their social security number (Clinic 3) Did not write social security number on urin cultivation kits (Clinic 5)
9. The patient health record, data safety and confidentiality	Insufficient patient record keeping (Clinic 5)
14. Hygiene	Inadequate dishwasher (should be replaced) / incorrect use of desinfection fluids (Clinic 2) Insufficient use of the the autoclave (Clinic 2) Insufficient use of soap in the sterilisation process (Clinic 5) Use of normal oven insufficient for sterilisation of instruments (should be replaced with an autoclave) (Clinic 5) Did not have an apron for use with contagious patients (Clinic 5 and 10)

The surveyors were in agreement with IKAS on having a trust-based approach in their meeting with the clinics and they described how trust in the written and oral statements of the professionals was a fundamental part of the survey visit and their assessment. Trust was both something they valued and something they viewed as a necessity.

Further, the surveyors stated that they had rarely found reason to mistrust what the professionals told them and that the professionals at several occasions had disclosed information which showed that aspects their practice was not in concordance with the standards:

“We trust that people want this enough so that they will be honest about it. And sometimes we also experience that the GPs are totally honest and tell us that they are not doing things [correctly] […] where I think, oh, if you had just expressed yourself a little differently, I would have believed that everything was all right [laughing]” (Staff-surveyor #2).

They also believed that due to their techniques and surveyor-experience, they were quite good at assessing the reliability of the professionals’ descriptions:

“Well, I have become good at reading people by now, uh, so, or if they say something in an uncertain way or if it is incoherent and things like that, you see, but anyway, it is extremely rare that I have experienced that” (Staff-surveyor #3).

However, the surveyors were aware of the possibility of the professionals not being completely honest, and that they, especially in certain areas, had little chance of detecting if the descriptions of their procedures were not in accordance with actual behaviour. Instead, they had to take the professionals’ descriptions for granted:

“I can’t check it, because if they tell me that they clean [the clinic every day], well then I have to trust it” (Staff-surveyor #3).

In the few cases where they suspected that the descriptions were not completely truthful, their approach was to’drill deeper’ into the issue. If that was insufficient, the surveyors would make a point of explaining the importance of complying with the standards and sometimes describe how they also had to make adjustments in their own clinic in order to comply with the standards.

### The professionals’ approach, experiences, and reflections

#### Generally striving for compliance with the standards

Although the professionals’ attitudes towards accreditation varied substantially from quite positive to quite negative, they had generally sought to comply with the standards prior to the survey. An exception to this was a GP who had decided that the clinic should only work with a few of the standards prior to the visit, and then make additional changes after the visit based on the surveyors’ assessment.

The professionals had different motives for striving for compliance with the accreditation standards. In a majority of the clinics, they found that most of the standards were meaningful and that the clinic was already in compliance (or nearly in compliance) with many of the standards. Several of them expressed that going through the accreditation was a task that had to be completed like other external requirements. The most positive minded saw accreditation as an occasion to critically assess and develop their own clinic and wanted to do everything according to the standards. For some, it was also a question of pride and reputation. Others just wanted to be sure to pass accreditation in the first attempt in order to avoid spending additional time on preparing for and going through an additional survey visit. Further, several of the professionals had little knowledge of how the survey visit would be conducted and what kind of approach the surveyors would take. Prior to the survey, most of the professionals portrayed the survey visit as an exam with a focus on control, and while some felt confident in succeeding, others were quite nervous in advance. The uncertainty about what to expect led some professionals to work more extensively with the standards.

The professionals’ approach to the survey visit was generally to describe the structure and activities of the clinic in a manner that corresponded to actual practice:

“We just have to answer straight from the heart, right? It is not about remembering what to say, you see [laughter]. We just have to tell what we do” (Clinic #4, Staff).

Several of them explained that they had nothing to cover up or hold back since they felt well-prepared believing that everything was consistent with the standards, and since deception was not an appropriate option for them:

*“I don’t think we had a need to cover up things. I don’t think so because things were as they should be, you see. Uh so, we would not, i.e. we told things as they were, you see. I think we will always do that. Well, I actually think it is like that. To me it is a fundamental thing, you see” (Clinic #1, GP).*

However, since the professionals perceived the survey visits to be an examination, they generally did not just speak bluntly and had been carefull about how they described their procedures in order to prevent misunderstadings and to make sure they were assessed correctly. A few GPs also commented that had it just been a normal meeting among collegues, then more details would have been provided:

*“I think we were all in a state of alertness, I mean we were attending an examination. Therefore, things should be said properly, and, and you should be able to vouch for it, but we said it in such a way that it was presented in the best possible manner. (…) So of course, I didn’t just speak out bluntly” (Clinic #1, GP).*

As mentioned in the section above, the GPs had to show the surveyors some examples of patient records during the survey. In most of the clinics, the GPs arbitrarily retrieved the patient records during the survey visit but in three of the clinics, the GPs had selected the patient records in advance. One of the GPs expressed that she had selected patient records for patients whom she expected to be well-managed (although also stating that she believed most of her patients to be so). However, it turned out that one of the selected records had some flaws. The two other GPs had not selected records for well-managed patients but records that illustrated their workflow, for patients whom they knew well, and for patients where there was something to talk about. Hence, the GPs did not seem to be particularly tactical in their approach to selecting patient records.

#### Misrepresentation as a rarely used tactic

According to the professionals they had generally tried to give an authentic portrait of the clinic to the surveyors. However, a few cases of misrepresention did surface in our interviews (see Table 6). When misrepresentation occurred it was limited to one or two standards where the professionals disagreed with some of the reqiurements. Thus, several of the professionals deemed that some requirements were too excessive (such as those included in the standards on hygiene and secure identification of patients), and while some chose to comply anyway, these were the areas where a few clinics considered non-compliance and engaged in misrepresentation. They could either misrepresent when describing their activities in relation to the standards, or they could manipulate certain visual impressions given to the surveyors in order to avoid questions about specific issues. For example, in one clinic where the GP and the nurse disagreed with some of the hygiene requirements (concerning cleaning of toys in the waiting areas and daily cleaning of the clinic), they reported to the surveyors that they adhered to the requirement although this was not so. This GP had heard from several of her colleagues that they would tell the surveyors that their clinic had cleaning every day even if this was not the case. Therefore, this GP was afraid that she would stand out negatively from the rest if she did not do the same:

“[originally] I did not intend to say that we had cleaning more than twices a week…sometimes a bit more if it is neccesary… [but] here I compromised on my principles because I did not have the energy to do otherwise […] It was an area where I considered that the accreditation was too excessive and that it was not fair” (Clinic #10, GP).

**Table 6:** Cases of misrepresentions during the surveys and at folllow-up

Clinic	Misrepresentions
Clinic 3	Most of the professionals in the clinic did not reveal that they did not ask all patients for social security number, but occasionally used visual recognition. While one GP revealed this at the survey, he subsequently misrepresented their procedures in the follow-up call with the accreditation agency.
Clinic 5	• Did a thorough cleaning of the clinic right before the survey visit to ensure that the clinic appeared impeccable • The clinic had been given remark for not keeping sufficiently detailed patient records, but the GP intended to stay non-compliant in this area and did not disclose this at the survey visit Although the GP expressed intentions to change procedures for COPD care in response to the remarks of the surveyors, he had not done so at the time of our second research interview.
Clinic 7	GP disagreed with the requirements of always wearing short sleeves (which she did not usually do) but during the survey visit she would wear short sleeves. For some patients, the GP ensured patient identity only by visual recognition (and not through oral confirmation of social security number) but did not reveal this at the survey.
Clinic 10	The professionals had agreed to say that they adhered to the standard concerning cleaning of toys in the waiting areas and daily cleaning of the clinic although this was not entirely true. The GP did not label testkits with social security numbers, but placed the test sample and patient information together in a box for the nurse to complete. Nurse had taken of her rings of a couple of days before the survey visit in order to avoid questions about this.
Clinic 12	Did a thorough cleaning of the clinic before the survey visit although this was not usual practice.

The few cases of visual misrepresentation included professionals taking off jewelry before the visits and wearing short sleeves instead of their usual long sleeves. In this way, they could avoid questions about these issues, which they considered to be of minor importance in general practice.

As described in the previous section about the surveyors, some inconsistencies between the standards and usual practice in the clinics were identified at the survey visit. In most clinics that got a remark for such inconsistencies, the professionals chose to implement the changes required to comply with the standards. Some had been unaware that the acitivities in question had not been in alignment with the standards, and some had awaited the surveyors’ interpretation of particular standards. However, in the follow-up process, the GPs from two clinics pretended that they would make changes to meet the requirement without having intentions to do so. In the first clinic, they had originally planned to misrepresent their procedures in relation to the requirement of always asking patients for their security number when performing tests (since the GPs found this requirement to be overkill). However, during the survey visit one of the GPs admitted to the surveyor that they did not always do so if they knew the patient very well. When the clinic was given a remark for not complying with the standard, one of the GPs contacted IKAS after the survey visit and declared that they were now asking all patients for their social security number. The GP found this follow-up ridiculous because the clinic had not changed their procedures.

In the second case, a clinic had received remarks for not keeping sufficiently detailed patient records and for providing too unsystematic care for COPD patients. Here, the GP found that making the required changes in record keeping would be too troublesome and of little value in daily practice, and therefore he chose to stay non-compliant in this area, although he did not disclose this at the follow-up survey visit. Regarding COPD care, the GP expressed intentions to change procedures but had not done so at the time of our second research interview.

The cases of misrepresentation presented in this section emphasise that some of the accreditation requirements were controversial in general practice, and that, for some requirements, it was possible for the professionals to conceal non-compliance from the surveyors. While a few professionals had used misrepresentation as a supplementary tactic for achieveing accreditation without implementing all of the requirements, some professionals also described a less deliberate process where some of the activities which had been implemented stringently to comply with the accreditation standards were enacted less systematically in the time after the survey visit. These were usually activities that the professionals deemed to be of little clinical significance (such as a reduction in the frequency of the logging of performed controls, less frequent cleaning of e.g. desks and couches, less frequent identification of patients by social security number, and slight readjustments concerning follow-up on paraclinical tests).

#### Survey visits generally seen as sufficient for assessing compliance

The professionals from the clinics all described the atmosphere during the survey visit as pleasant, the conversation as relaxed, and the surveyors as kind. The visit was less interrogating than some had expected and more conversation like. They also deemed that it was important that the surveyors were colleagues because they believed that the surveyors’ understanding of their working conditions ensured a more proper assessment of their clinics (than if the surveyors did not have experience from general practice).

In spite of the above mentioned examples of misrepresentation, the professionals generally considered that the surveyors got a realistic picture of their level of compliance with the accreditation standards. While several of them found that the survey visit had been less thorough than they had expected, most still found that the surveyors’ way of questioning, combined with their visual impression of the conditions in the clinic as well as the surveyors’ experience and professionalism, was sufficient to ensure that the various standards were well covered:

*“The [doctor-]surveyor was proficient enough so that he would ask into things, and when he sensed that it was okay he went on to the next issue. So it went smoohtly, you could sense that he had a good feeling about how things should be” (Clinic #1, GP).*

However, a few of the professionals described the visit as too superficial for a proper assessment. They found that the survey-interview had been too much of a ‘chit-chat’ and especially that the assesment of the patient records had been too superficial. Further, some explained that there were areas in which compliance could have been visually assessed, but had not been so (e.g. checking quality controls of equipment, certificates from education, the actual refrigerator temperature, log-schemes, the emergency bag, and looking further back in the patient records). Some of them assumed that if the surveyors had not been particularly detailed it was because they had been satisfied with the documents sent forward prior to the visit and/or because they had gotten a positive impression of the clinic early on in the survey visit. In addition to this, a few professionals described that there were areas where they were not compliant with the standards which they had not been questioned about during the survey visit (e.g. the toys in the waiting areas being washable and wearing short sleeves).

## Discussion

In accreditation, the survey visit is the key method for assessing compliance with the pre-established standards. In our study, the surveyors combined obtainment of documents, questioning of the professionals, and visual observations to produce information on which to base their assessment. They sought to de-dramatise the survey visit, to generate a natural conversation with attention to workflows, and to use of the various spaces of the clinic as reference points during the interview. Their level of scrutiny was influenced by the quality of the documents they had received from the clinics and by the observations they made in the clinic during the visit. They had a high degree of trust in the professionals, and believed that the information they obtained was sufficient to ensure a valid assessment of the clinics’ level of compliance with the standards. According to the professionals they had generally presented the surveyors with an authentic portrait of their clinic and had mostly sought to comply with the standards. Still, misrepresentation was employed a few times due to disagreements with the standards.

The surveyors had been attentive towards establishing a good relationship with the professionals from the first contact and believed that their status as also being colleagues contributed to this. According to the surveyors, the clinics were generally well-prepared for the survey visit and both the surveyors and the professionals reported that the surveys had been carried out in a positive atmosphere. This corresponds to a recent study of surveyors’ perceptions of their data collection opportunities where staff engagement was experienced as an important facilitator for performing accreditation surveys [18]. And, in contrast to the results reported by Greenfield et al. [6], we did not find that difficult relationships between the surveyors and professionals made the professionals hide or withhold information from the surveyors. As mentioned, the few cases of misrepresentation identified in this study were more related to the professionals questioning the meaning and value of some of the standards.

In accordance with the accreditation agency’s directions, trust played a central role in how the surveys were performed. The literature on trust differentiates between a) a general propensity to trust and b) a more situation-specific trust [19], and in the surveyors’ approach we can identify both. Propensity to trust is not based on concrete actions by another person and is present prior to availability of data. The propensity to trust is sometimes seen as a personality trait but according to the literature it can also be influenced by considerations of the trustworthiness of the other party, e.g. by beliefs about professional competences and integrity [19]. We found that the surveyors met the clinics with a permeating sense of trust, which was influenced by a belief in the professionals’ intentions to comply with the standards due to professionalism and integrity. Further, our data suggest that the surveyors also build up what can be described as an experienced based propensity to trust, meaning that their general sense of trust in the clinics was reaffirmed over time because they rarely found reason to believe that the trust had not been warranted. We also identified a more situation-specific trust. Here, the surveyors gained an increased sense of trust in the specific clinic’s performance and compliance based upon what they read in documents prior to the survey, on selected visual observations, or on the responses to questions asked during the survey. Both the general propensity to trust and the situation-specific trust seemed to influence how the surveyors conducted the visits in terms of the areas covered and the depth of inquiry. For example, the high degree of trust implied that the surveyors at times relied strongly on the accounts of the professionals rather than on using the available visual opportunities (e.g. checking expiration dates on medicines and utensils, looking at equipment and annual plans for control, and more extended use of the information contained in the patient records). Hence, it is important to consider if high levels of trust in one data source might reduce the use of other data sources in the assessment and thereby undermine the idea of using data triangulation in accreditation survey visits [6].

The surveyors generally believed that they were able to obtain a valid impression of the clinics’ level of compliance with the accreditation standards. In a study of external reviews at hospitals, Walshe et al. [20] also found that members of the assessment team had a strong confidence in their own ability to obtain a true picture of the activities under review. Still, our study also showed that the (few) instances of misrepresentations, which we identified, were not detected by the surveyors, indicating that this level of confidence is not always warranted. It also questions whether the surveyors would have been able to identify more profound levels of noncompliance if the professionals had been less willing to comply with the standards.

In the mandatory Danish accreditation programme, compliance could only be assessed visually for some standards; for other standards, the surveyors had to rely on the statements of the professionals during the survey visit (e.g. the use of social security numbers, cleaning, and procedures for managing a selected vulnerable patient group). It may seem paradoxical to base the assessment of compliance with externally imposed standards on such a high degree of trust in the assessed actors, and it could be argued that only standards that can be visually assessed should be included in mandatory accreditation programmes. On the other hand, such an approach may result in a too narrow perspective on clinical practice. Further, the results from the present study suggest that a trust-based approach can be relevant if the professionals generally agree with the content of the standards. In that case, they will generally seek to comply with the standards, and the trust-based survey visit will then be able to identify deviations from the standards caused by a lack of understanding and knowledge. Thus, it seemed to be an advantage of the process questions used by the surveyors that they can expose misunderstandings and shortcomings which the professionals are not aware of. Also, while misrepresentation is still possible, it may likely be more difficult to do in a coordinated way when talking about complex processes. Conversely, the results also suggest that the validity of a trust-based survey visit may be threatened if the professionals are generally in (strong) disagreement with the standards (or, although not evident in our data, if the professionals, despite finding the standards reasonable, have been unable to implement them due to lack of resources or time, but still wish to receive accreditation without further effort). In such cases there might be a higher risk that the professionals will engage in misrepresentation which will then go unnoticed if the assessment of compliance primarily relies on self-reporting. This suggests that for a trust-based approach to accreditation, it is not only crucial to develop standards in collaboration with the representatives from the profession but also to work on convincing the rest of the professionals of the merits of the standards prior to the survey visit. Alternatively, supplementary methods for investigating compliance could be considered such as use of register data or unannounced visits. However, we are not aware of studies from general practice documenting the value of such methods in terms of assessing compliance with accreditation standards.

### Strengths and limitations of the study

It is a strength of the study that we interviewed the professionals in the clinics twice and observed their survey visits. In the first interviews we obtained knowledge of their perceptions and intentions unbiased of their experiences at the survey visits and we could use this knowledge and our observations to formulate the questions for the second round of interviews. However, it is a limitation of the study that we (like the surveyors) relied on interviews with the general practice professionals about their behaviour in daily practice in relation to the accreditation standards rather than on observations of that behaviour. Thus, we cannot refute that they might have misrepresented their behaviour in the research interviews just as they might have done at the survey visit. Thus, the levels of misrepresentation and noncompliance with the standards might have been more profound than our results suggest. Further, since there were only few examples of misrepresentation among the clinics in the study, we had limited data of the surveyors’ abilities to uncover intentional non-compliance. Finally, although the professionals reasoned that their non-compliance was in areas of less importance, the actual impact hereof could not be assessed in this study.

## Conclusion

Although the survey visit is the central assessment method in accreditation, few studies have investigated how information about compliance with the accreditation standards is produced by professionals and surveyors in relation to the visit, and how the actors experience the visit as a method for assessing compliance. In this study of accreditation in Danish general practice, trust in the professionals’ self-reported behaviour played a crucial role in the surveyors’ approach to the survey visits. Hence, the validity of the survey visit as a method to assess compliance was highly dependent on the professionals’ willingness to convey a realistic picture of their practice. The results suggest that they were generally willing to do so, and that the trust-based approach was suitable for identifying non-compliance caused by a lack of understanding of the individual standards among the professionals. The study also indicates, however, that some professionals may engage in misrepresentation of their practice if they are in strong disagreement with the accreditation standards, and that such misrepresentation is difficult to detect for the surveyors. Thus, when adopting a trust-based approach to the survey visit (i.e. where the assessment of compliance primarily relies on self-reporting), it seems particularly important to ensure that the professionals view the standards as meaningful and manageable.

## Supplementary Information

**Additional file 1.** Interview guides.

## Data Availability

The anonymised transcribed interviews from the current study are available from the corresponding author upon reasonable request.

## Abbreviations

GP: General practitioner
IKAS: Danish Institute for Quality and Accreditation in Healthcare
