# Supplementary material for: The survey visit as a key evaluative event in accreditation–a qualitative study of survey visit experiences among surveyors and general practice professionals
Source: BMC Fam Pract. 2021 Jul 31;22:163. doi: 10.1186/s12875-021-01497-7 (PMC8325228; doi:10.1186/s12875-021-01497-7)
Supplement: Supplementary file 1 — Additional file 1. Interview guides. [file 12875_2021_1497_MOESM1_ESM.docx]

# Additional file 1: Interview guides

In the following we present a translated version of the interview guides used in the study. Please note that only some of the questions were relevant to the research question addressed in the paper at hand. Other questions are related to the issues explored in our two previous papers^[[1]](#endnote-1)^. Also, please note that the interview guides were modified during the data collection process based on the experiences from previous interviews. Further, the semi structured interviews were flexible and dependent on the responses of the informants. Dots indicate potential follow-up questions.

| **First interview with the clinic** | | |
| --- | --- | --- |
| **Introduction** | 1. Information about the interviewer, the research project, confidentiality and anonymity. 2. Information about the structure of the interview. 3. Questions about the clinic’s organisation, staff, and those present at the interview. | |
| **Coherence** | 1. What did you think when you first heard that general practice was to be accredited?  - Thoughts after having read the standards and started working with them.  1. Why do you think that the public health authorities have decided that general practice should be accredited? 2. What do you see as the purpose of accreditation?  - Thoughts concerning the discussion on control vs. quality development  1. Do you find that GPs and staff in the clinic are in agreement considering your views about accreditation and how to work with it? (please elaborate…) 2. Do you have a clear understanding of the individual standards?  - Are some standards more difficult to understand than others? (please elaborate…) - Do you have a clear understanding of what is needed before you can be accredited? [in general and in the individual standards] (please elaborate…) - Does the clinic have a common understanding of the individual standards and what it takes to live up to them? (please elaborate…)  1. Do the standards indicate a different way of doing things than usual? (please elaborate…) 2. Assuming that you live up to all the standards when you finish this process - how do you think it will affect the quality of your services in this clinic?  - Why / how / why not?  1. What is your assessment of the professional relevance of the standards?  - The relevance to general practice as a whole - The relevance for your clinic - The relevance of the indicators in the individual standards  1. What are your thoughts about the basis of the standards?  - In relation to professional knowledge and evidence  1. Do you see accreditation as a tool for quality improvement? (please elaborate) 2. How does the rest of the clinic [GP and staff not present in this interview] perceive accreditation? | |
| **Cognitive Participation** | 1. How would you describe your approach to working with accreditation?  - To be accredited with the least possible effort, seeing it was an occasion to develop your clinic, checking what you do is consistent with the standards/ is good enough. - Has your approach changed during the process? (and if so, how?)  1. Up until now, how have you been working with the standards?  - Who took the initiative? - What meetings have been held (and what was their content?) - Division of tasks – the role of the staff  1. How much time have you spent understanding and working with the standards? (please elaborate) 2. Have you participated in the support activities arranged by the region (information meeting, workshops)?  - Content and usefulness of activities - Reason for not participating in support activities | |
| **Collective action** | 1. What tasks have each of you carried out as part of the work of meeting the standards?  - Have you read/re-read guidelines and disease management programmes in connection with working with the standards (e.g. COPD and diabetes)?  1. How have you experienced working with the standards?  - Which standards are (have been) easiest to adhere to / to implement and why? - Which standards are (have been) the most difficult to adhere to and why? [In case the informants have difficulties recalling particular standards, ask about standards that other informants have mentioned as problematic for example hygiene and test results)] - How have you dealt with the challenges?  1. Are there any particular conditions in your clinic that have facilitated your work with the standards? (please elaborate) / Are there any particular conditions in your clinic that have impeded your work with the standards? (please elaborate) 2. Have you used the DAK-E system?  - For what and how has it worked?  1. Have you sought out any of your colleagues for advice or inspiration while working with the standards?  - In what way and experience of benefits and limitations  1. Have you been lacking any competencies while working with the standards (IT, project management etc.)? (please elaborate) 2. Have you produced written documents of your procedures as part of the accreditation process?  - If yes, which ones? - What does it mean for you to have the procedures in writing?  1. Have you performed the required journal audit, the patient evaluation, and chosen a vulnerable patient group for a targeted effort?  - Reflections about these mandatory requirements.  1. Are there things you already do differently than before due to having worked with the standards? [or is it rather existing practices that are now put into writing?]  - Please elaborate on any current or expected changes to practice  1. Are there standards that you have chosen not to adhere to? 2. Did you have sufficient time to work with the standards and ensuring adherence? (please elaborate)  - What are your thoughts about the financial reimbursement in relation to accreditation?  1. How has working with the standards been compatible with other quality improvement processes in the clinic? | |
| **The survey visit** | 1. How is your approach to working with the standards influenced by the fact that you receive a visit from a surveyor who will assess adherence to the standard? 2. What thoughts do you have about the survey visit? 3. How will you prepare for the visit? 4. What are your thoughts about the surveyor being a colleague [the GP and in some cases the staff]? 5. Do you expect to be accredited? (please elaborate)  - What would it mean to you, if you were not accredited? | |
| **Closing** | 1. Is there something you want to add, something important we have not talked about concerning your process or the specific standards? | |
| **Second interview with the clinic** | | |
| **Introduction** | | 1. Information about confidentiality and anonymity 2. Information about the content and structure of the interview |
| **Process after first interview** | | 1. What was your process in the clinic between our first interview and your survey visit?   Follow up questions specific to each clinic about their process before the survey visit based on the first interview.   1. …. 2. …. 3. ….     Follow up questions to all clinics about the process before the survey based on the first interviews.   1. Was it easy or difficult to find out what it would take to adhere to the standards? (please elaborate)  - For which areas/standards could a higher degree of specificity have been beneficial, e.g. a checklist?  1. What material did you read while working with the standards?  - Where else did you find information?  1. Last time you told us that you had used the written examples of procedures as an inspiration - can you elaborate on how you used them more specifically? 2. One clinic in this study had received support from a regional consultant – have you heard about this option, considered using it, or at other times contacted the region or IKAS with questions?  - Reasons for use / non-use  1. Do you expect to use the descriptions of your local procedures yourself, or are they mainly used to document that you adhere to the standards?  - Were they written differently, because they were to be used for accreditation and uploaded to IKAS (formulations, structure etc.)? (please elaborate)  1. Did you feel ready for the survey visit? (please elaborate) 2. With the knowledge you have now (after the survey) is there something you would have done differently in the process before the survey? (please elaborate) |
| **The survey visit** | | 1. How would you describe the survey visit? What was your overall experience of the visit? 2. What do you think about the way the surveyors tried to clarify whether you adhered to the standards?  - Was it more or less detailed than expected? - Do you think the way the surveyors questioned you was appropriate to assess whether the clinic adhered to the standards? Do you think the way the surveyors questioned you was appropriate to assess the quality of what you do in the clinic? - Were there any important areas the surveyor did not get into? (please elaborate)   How will you describe the interaction with the surveyors during the survey? (please elaborate)   1. To what extent where you mindful about what you told the surveyor?   [e.g. cautious not to say something wrong, not saying more than necessary, , saying what they wanted to hear, answering openly on all issues]   1. To what extend can the surveyors know whether the things you tell them are true? (please elaborate on your thoughts about this) 2. Was it your impression that the surveyor had read the documents you had uploaded - how were they included in the survey visit? 3. How did the visit fit with your expectations?  - Was there anything about the visit that surprised you? - Did you expect that there would be areas where the surveyor would be critical about you current practice / areas where you would receive remarks?  1. How would you primarily describe the survey visit: As a control visit? Or as an opportunity to get inspiration or advice from a colleague? (please elaborate) 2. [To those clinics who received remarks] - what do they think about the remark(s) you received? 3. After the visit, do you think that there are areas where you have done too much? (over-implementation) |
| **The time after the survey** | | 1. How has the process been after the visit?  - Have you changed anything after the visit? (please elaborate) |
| **Impact** | | 1. What do you think it has meant for your clinic that you have been through the accreditation? 2. If we try to go through the individual standards, can you tell me if you are doing something different now? (show overview)  - Small changes versus more significant changes - [For those who mention or have previously mentioned that the accreditation process has given increased systematics] - what does this mean and entail? - Did your discussions about procedures in the clinic have a practical impact? (please elaborate) - Did writing down your procedures have a practical impact? (please elaborate) - Has there been an impact of you knowing more about what the others in the clinic are doing? (please elaborate)  1. Are there any changes that you have already reversed? Or that you think will be reversed in the future? 2. Has it improved the quality of your work that you have been through the accreditation process? - please elaborate in which way. 3. [To the predominately positive informants: Was there anything negative about the accreditation process? (please elaborate) / [To the predominately negative informants: Were there any benefits of the accreditation process? 4. Do you think that the work with the accreditation has been worthwhile? (time investments vs perceived benefits) 5. Do you think that the work with the accreditation had any negative consequences? (please elaborate) |
| **Perspectives** | | 1. If there is another round of accreditation, what do you think the requirements should be? And what should be done differently? 2. The changes that accreditation has brought about in your clinic: Do you think they could have been achieved in other ways? 3. Some clinics have mentioned in previous interviews that there are some clinics that need a quality boost. Do you think that accreditation is a suitable method to achieve this? - If not what could be done instead? 4. And more broadly for general practice: Do you have any suggestions for how to ensure the quality of general practice in the future? (methods and which areas to focus on) |
| **Interview with surveyors** | | |
| **Introduction** | | 1. Information about confidentiality and anonymity 2. Information about the content and structure of the interview |
| **About the surveyor** | | 1. Why have you chosen to be a surveyor? 2. How many surveys have you completed so far - and how many are you going to do in total? |
| **Before the visit** | | 1. Can you describe your communication with the clinics before visits?  - What about the clinics: what do they ask about?  1. How do you use the documents the clinic has uploaded in advance (guideline from IKAS or own approach?)  - Do you make a strategy for the visit based upon this?  (focus areas, approach etc.) |
| **Approach at the survey** | | 1. How would you describe the way you approach the visits? 2. Can you elaborate on the reasoning behind your approach / your method of questioning?  - Some have talked about getting a quick sense of the clinic and then ask if they notice problem areas - what do you think about it? - Use of tracing / course questions - when and experience with that technique - Do you have a mental list with core checkpoints when reading documents and when conducting the visits? - GP-Surveyor: What are you looking for in the medical record system? What are your thoughts about the clinic selecting patient cases themselves? - Differences and similarities between the approaches in different clinics - what determines this? - Thoughts about some clinics describing that clinics are assessed differently (depending on the surveyor and over time)  1. Did the surveyor training make you feel well-prepared for the role? |
| **The standards** | | 1. What do you think about the relevance and the level of the selected standards and indicators? 2. Is it clear for all the standards / indicators whether the clinics adhere to them or not? (please elaborate)  - Standards easy / difficult to assess - Examples of the surveyors discussing whether a clinic adhere to the standards and perhaps disagree. - Have you contacted IKAS during a visit for with questions about an assessment? - Degree of judgement involved in the assessment - examples  1. Which standards are most often problematic in terms of – adherence and understanding? Reflections on and experiences with specific standards mentioned by the clinics:  - Hygiene standard (clinics experience lack of clarity, doubt) - Patient identification - requirements and how it is assessed  1. Have there been any changes in the requirements during the accreditation process? Which ones? 2. What are your thoughts on whether indicators could be formulated more clearly? 3. How do you see your options in terms of getting a solid impression of whether the clinics adhere to the standards? What are your thoughts about the fact that the clinics might in principle come up with descriptions of their practice (in writing or during visits and follow-up) that do not correspond to what they actually do? |
| **The surveyor as a colleague** | | 1. Challenges and benefits of being a colleague (to co-surveyor: that they are / are not colleagues)  - Balancing the role of an IKAS representative with being a colleague - Do you use the fact that you are a colleague actively during the survey - Considerations about talking about their own clinics  1. How do you experience that the clinics see you? |
| **Surveyor and co-surveyor** | | 1. Please describe the role distribution between surveyors – does it vary between visits? (please elaborate) 2. Please describe the assessment conversation between the surveyors (negotiation, agreement, judgements) (plus any differences in judgements) 3. How would you describe the surveyor group?  - Do you experience that GP-surveyors and co-surveyors differ from each other? |
| **About the visits in general** | | 1. What is your overall experience of the visits? 2. How do you experience being received by the clinics? 3. Challenges when visiting  - Have you experienced any clinics that were negative about the visit? What have you done in such cases? - Are there cases where you have assessed in advance that the clinic cannot pass the accreditation based on the documents? What do you do in such cases? Examples of visits where things have not gone so well - Clinics not being accredited - Disagreement between the two surveyors  1. What do you do if you experience that something seriously wrong? – (reporting obligation) |
| **The specific visit we observed** | | 1. … 2. … 3. … |
| **Accreditation as a method** | | 1. What do you consider to be the purpose of accreditation? 2. How would you describe the impact of accreditation in the clinics?(please elaborate)  - Does it make sense that all clinics must go through accreditation to find those with problems? (please elaborate)  1. Have you experienced cases of over-implementation in the clinics? (please elaborate) 2. Would you sign up as a surveyor in case of a second round of accreditation- reasoning? (please elaborate) |

1. Due TD, Thorsen T, Kousgaard MB. Understanding accreditation standards in general practice - a qualitative study. BMC Fam Pract. 2019 Jan 31;20(1):23. doi: 10.1186/s12875-019-0910-2.

   Kousgaard MB, Thorsen T, Due TD. Experiences of accreditation impact in general practice - a qualitative study among general practitioners and their staff. BMC Fam Pract. 2019 Oct 28;20(1):146. doi: 10.1186/s12875-019-1034-4. [↑](#endnote-ref-1)
